# Modeling early haematologic adverse events in conformal and intensity-modulated pelvic radiotherapy in anal cancer

**DOI:** 10.1016/j.radonc.2015.09.009

**Published:** 2015-11

**Authors:** Maxwell Robinson, Ahmed Sabbagh, Rebecca Muirhead, Lisa Durrant, Frank Van den Heuvel, Maria Hawkins

**Affiliations:** a Department of Oncology, University of Oxford, UK; b Department of Clinical Oncology, Oxford University Hospitals NHS Trust, UK

**Keywords:** Haematological toxicity, Pelvic bone marrow, Intensity modulated radiotherapy, Normal tissue complication probability modeling

## Abstract

**Background and purpose:**

To determine if there are differences between dose to pelvic bone marrow (PBM) using intensity modulated radiotherapy (IMRT) under UK guidance versus conformal radiotherapy (CRT) per ACT II protocol and if differences translate to rates of early haematological adverse events grade 3 or greater (HT3+).

**Methods and materials:**

Two groups of 20+ patients, treated under IMRT and CRT regimes respectively, were identified. All patients underwent weekly blood cell count: haemoglobin (HgB), white cell count (WCC), absolute neutrophil count (ANC) and platelets (plats).

Percent volume of PBM and sub structures receiving 5–25 Gy were tested for statistical significance. Regression models were used to test for correlation to blood counts. NTCP modeling was also performed.

**Results:**

PMB dose metrics showed a significant increase in the IMRT group. Regression analysis showed iliac and lumbosacral PBM dose metrics to associate with reduced nadir ANC and WCC. NTCP at HT3+ was 0.13 using IMRT relative to 0.07 using CRT (*p* < 0.05).

**Conclusion:**

Whilst this is a relatively small retrospective study and lacks information on the distribution of active PBM, IMRT treatment has been shown to significantly increase PMB irradiation. PBM dose metrics have been shown to be predictive of WCC and ANC suppression. NTCP modeling predicts much high risk of HT3+. Paradoxically, actual rates of HT3+ were comparable suggesting that differences in the distributions of dose metrics maybe a significant factor and/or that there are insufficiency in the NTCP modeling.

Radical chemoradiation is the standard treatment in loco-regional anal cancer, achieving a 3 year disease free survival of 73% with organ preservation [1], [2], [3]. Recently, there has been an increasing move from conformal to intensity-modulated radiotherapy (IMRT). IMRT can deliver varying dose levels to multiple targets while decreasing low to intermediate dose (V30 Gy, V40 Gy etc.) to organs at risk, reducing adverse events (early gastrointestinal and dermatological, grade 3+) [4]. However, the impact of IMRT in delivering a ‘low dose bath’ to normal tissue needs to be considered in this tumour type, particularly in the context of concurrent chemotherapy. This impact is most pronounced in highly chemo-radiation sensitive tissue such as bone marrow (BM). Increased irradiation of BM has been shown to increase likelihood of early haematologic adverse events (HT) [5], [6]. Approximately one third of proliferating bone marrow is located in the pelvic bones [7]; therefore differences in delivery system in anal irradiation may result in significant changes in rates of early adverse events. Irradiation of BM in IMRT can be reduced by applying appropriate dose objectives in the optimisation, i.e. through sparing [8], but this is not routinely done during the planning process.

UK ACT II trial delivering a 2 phase conformal radiotherapy (CRT) technique reported early haematologic adverse events grade 3 or 4 (HT3+) in the mitomycin arm as 26% [2]. The US Radiation Therapy Oncology Group (RTOG) 98–11 trial reported maximum grade 3 of 35% and max grade 4 in 26% of patients recruited in the mitomycin arm [3]. The RTOG 0529 trial looking at IMRT for anal cancer reported 58% HT3+ (4). Other US IMRT studies which have reported HT3+ at 24 and 53% [9], [10].

This study aims to determine differences in pelvic bone marrow (PBM) dose associated with IMRT per UK guidelines (IMRT group), relative to CRT per ACT II (CRT group), and implications on acute haematological toxicity.

## Methods and materials

### Patient selection

Twenty-five and twenty-one anal cancer patients treated with CRT and IMRT chemoradiotherapy respectively were identified in this retrospective study. Patients were treated in two sequential blocks in 2009 and 2014 respectively. Patients had a diagnosis of anal carcinoma with squamous carcinoma (one adenocarcinoma, IMRT group), disease localised to the pelvis, were radiotherapy naïve and considered fit enough with adequate baseline bloods by the treating clinician for radiotherapy alone or chemoradiotherapy. Patient gender, age, tumour staging and nodal status were collected.

### Treatment

#### Radiotherapy

Twenty-five patients were treated as per ACT II protocol [2], using a two phase CRT technique. Phase one was a two field (anterior-posterior parallel opposing) technique to 30.6 Gy in 17 fractions which covered primary tumour, anal canal and elective nodes, field borders were placed superiorly 2 cm above the bottom of the sacroiliac joints, inferiorly 3 cm below anal margin or 3 cm below the most inferior extent of tumour, laterally to femoral heads. Phase two was a standard three field CRT technique to 19.8 Gy in 11 fractions covering gross tumour. The gross tumour was delineated by the treating oncologist with a margin of 3 cm added to create the PTV. No constraint was placed on pelvic bone dose including dose to femoral heads.

21 patients were treated using 7–9 field IMRT in 28 fractions using simultaneous integrated boost. Delineation was as per UK guidance [11]. In summary, gross anal tumour plus a 2.5 cm margin received either 53.2 Gy (if T3 and T4) or 50.4 Gy (if <T3); the involved nodes plus a 2 cm margin received 50.4 Gy and the prophylactic nodes received 39.2 (12 patients) or 40 Gy (9 patients) due to the protocol being updated during the audit period. A constraint was placed on femoral head dose (dose to 50% less than 30 Gy, dose to 35% less than 40 Gy and dose to 5% less than 44 Gy) but dose to other pelvic bone structures was unconstrained.

#### Chemotherapy

Patients fit enough for concurrent chemotherapy were planned to receive either Mitomycin 12 mg/m^2^ Day 1 monotherapy if 5-fluorouracil was contraindicated, Mitomycin 12 mg/m^2^ and 5FU 1000 mg/m 2 Days 1–5 and 29–33. 1 patient had Mitomycin 12 mg/m^2^ Day 1 with Capecitabine 825 mg/m^2^ twice daily on all days of radiotherapy. The second course of 5-fluorouracil was reduced by 25 or 50% following any episodes of any Grade 3 non-haematological related toxicity such as diarrhoea or Grade 3–4 haematological toxicity. 5-Fluorouracil was withheld at Grade 4 non-haematological toxicity and Capecitabine was withheld with thrombocytopenia Grade 2 or neutropenia G3 or any Grade 3 non-haematological toxicity related to Capecitabine; until it resolved to G1 then restarted at the same dose or at a reduced dose.

### Bone marrow delineation

Pelvic bone marrow (PBM) was delineated using the external surface of bone and sub divided into three components; iliac BM, extending from the iliac crest to the superior edge of femoral head, lower pelvis BM, extending from the superior edge of femoral heads and including all pelvic bone as well as proximal femoral bone down to the level of and including the inferior ischial tuberosities, and lumbosacral BM, extending from the level of the superior border of L5 to the superior edge of femoral heads. Sub division of BM was based on previously published work by Mell et al. [5], [6]. The method of delineating BM using the external surface of bone is consistent with the Radiation Therapy Oncology Group (RTOG) 0418 clinical trial as well as the aforementioned previous published work [5], [6].

### Dose metrics

The percent volume of pelvic, iliac, lower pelvis and lumbosacral BM receiving 5, 10, 15, 20 and 25 Gy (V5–25) was extracted. IMRT and CRT group dose metrics were compared using a two tailed Mann–Whitney *U* test.

### Blood parameter analysis

Haemoglobin (HgB), white cell count (WCC) including absolute neutrophil count (ANC) and platelets (Plats) were determined from blood samples collected at baseline and weekly during radiotherapy. In addition to absolute counts, blood count ratios were calculated by dividing counts at each week by baseline count. Toxicity was graded using Common Terminology Criteria for Adverse Events, version 4.0. Maximum toxicity grading during radiotherapy was noted for each patient. Analysis endpoints were blood count nadirs, blood count ratio nadirs and whether a patient had experienced acute HT3+. IMRT and CRT group blood count nadirs were tested using a two tailed unpaired Student *t*-test assuming unequal variance.

Dose metrics were compared with analysis endpoints using univariate and multivariate linear and logistic regression models to determine if statistically significant correlation to decreasing nadir blood counts, both absolute and count ratio, and increasing HT3+ probability could be established. Covariants of female gender, age, T3/4 and node positive status were also compared using regression models. Additionally, weekly blood counts and count ratios were compared between groups using a two tailed unpaired Student *t*-test assuming unequal variance.

Significance testing using the Holm–Bonferroni method of correction for multiple testing was applied. False discovery rate was controlled at 5%. In regression analysis false discovery rate was controlled at 5% for analysis of whole PBM and each PBM sub structures dose metrics against each individual blood count for IMRT and CRT groups separately.

### NTCP modeling

Lyman–Kutcher–Burman (LKB) NTCP [12] modeling on PBM was performed using parameter value estimates and 95% CI taken from previously published work by Bazan et al. [13] which estimated HT3+ parameter values based on anal cancer patients receiving mitomycin plus fluorouracil (mitomycin 10 mg/m^2^ on day 1 and 29 with fluorouracil 1000 mg/m^2^ on days 1–3 and days 29–32). Constraining n to one (i.e. treating bone marrow as an entirely parallel organ), *m* was reported as 0.09 (95% CI, 0.4–0.3) and TD50 as 30 Gy (95% CI, 28–32 Gy). NTCP was calculated using Eqs. (1), (2), (3), (4) [14] where $v_{i}$ is the volume within dose bin $D_{i}$ at 0.1 Gy intervals and *e* is the number of fractions. The alpha beta ratio $\left(\frac{\alpha }{\beta }\right)$ of PBM was taken as 10 Gy n.b. In the case of CRT plan $D_{\mathrm{eff}}$ was the sum of $D_{\mathrm{eff}}$ calculated for each phase.

(1) $$\text{NTCP}={\left(2\mathrm{pi}\right)}^{-0.5}\int_{-\infty }^{x}\exp\left(-\frac{t^{2}}{2}\right)\mathrm{dt}$$

(2) $$x=\frac{D_{\mathrm{eff}}-\text{TD}50}{m\times \text{TD}50}$$

(3) $$D_{\mathrm{eff}}={\left(\underset{i}{\sum }{\left(v_{i}\times \text{LQED}2_{i}\right)}^{1/n})\right)}^{n}$$

(4) $$\text{LQED}2_{i}=D_{i}\times \frac{1+(D_{i}/e)/(\alpha /\beta )}{1+(2/\alpha /\beta )}$$

## Results

Mean age was 66 yrs (44–88), 27/46 were female, 23/46 were T3–4 and 19/46 were node positive, 13/46 were both T3–4 and node positive. A breakdown of patient characteristics including chemo regime in each group is shown in Supplementary Table 1.

With the exception of lower pelvis BM V25 all BM metrics showed statistically significant increase in the IMRT versus CRT group (*p* ⩽ 0.02). Table 1 shows all dose metrics in both groups.

Four cases of leukopenia/neutropenia and 1 leukopenia were reported in the CRT group (20% HT3+) relative to one leukopenia, one leukopenia/neutropenia and one thrombocytopenia in the IMRT group (14% HT3+). Mean baseline bloods, absolute and ratio nadir counts are shown in Table 2. Blood count nadir did not show statistically significant difference between IMRT and CRT groups.

Univariate linear regression of the CRT group dose metrics against bloods showed iliac and lumbosacral BM V5–25 to have statistically significant association to a reduced nadir HgB (*p* < 0.02). Lower pelvis BM V5–25 showed statistically significant association to a reduced nadir Plats (*p* < 0.01). Lower pelvis V5–15 showed statistically significant association to a reduced nadir WCC and ANC (*p* ⩽ 0.03) but not after multiple testing was accounted for. Iliac V5–25 showed association to nadir WCC only (*p* < 0.05). All other dose metrics failed to demonstrate statistical significance. Univariate linear regression of the IMRT group dose metrics against bloods also showed iliac BM V15 and lumbosacral BM V10–25 dose metrics to have statistically significant association to a reduced nadir HgB (*p* < 0.05) but both failed significant testing after multiple testing was accounted for. Iliac V25 was suggestive of an association to reduced nadir WCC but it was not significant (*p* = 0.09). Lower pelvis V5–15 were not shown to associate with nadir WCC or ANC, however average V15 was 99%. All other dose metrics showed statistically insignificant association to reduced nadir bloods. Table 3 shows significant regression analysis result.

Weekly blood counts were not statistically significantly different in the IMRT and CRT group at any week. In both groups nadir counts occurred at week 2–3 followed by a recovery at week 4.

Univariate linear regression analysis of nadir absolute blood counts with covariates showed female gender to be a statistically significant association to reduced nadir in all counts: Beta −0.89 and −0.7, SE 0.4 and 0.32 and *p* 0.03 and 0.03.

Multiple linear regression analysis did not show dose metrics to be significantly associated with nadir blood counts. Univariate and multi variant logistic regression analysis also did not show dose metrics to be significantly associated with HT3+ using either IMRT or CRT.

Blood count ratio nadir did not show statistically significant difference in the IMRT population relative to the CRT. Univariate linear regression of the CRT group dose metrics against blood count ratios is shown in Table 4. Iliac V5–25 was shown to be significantly associated to reduced WCC (*p* < 0.01) and suggestive of an association to reduce ANC nadir (*p* < 0.1). Lumbosacral BM V5–25 suggested an association to reduced WCC nadir only (*p* > 0.05 but <0.06). Iliac V5–25 dose metrics were associated with reduced plats ratio (*p* < 0.02). Univariate linear regression of the IMRT group dose metrics against blood count ratios showed iliac V5–20 to be significantly associated to reduced WCC nadir (*p* < 0.01) with V10 and 15 suggestive of an association (*p* = 0.053 and 0.048) to nadir ANC. All lumbosacral dose metrics were significantly associated to both reduced WCC and ANC nadir (*p* < 0.05).

**Fig. 1:**
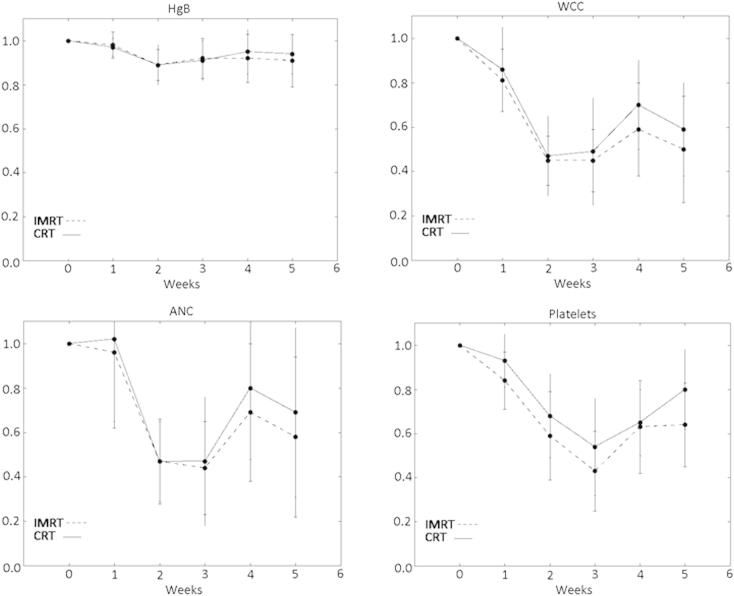
Weekly blood cell counts expressed as a ratio of baseline (mean and standard deviation) during radiotherapy showing intensity modulated (IMRT) versus conformal radiotherapy (CRT) sample populations. HgB: hemoglobin, WCC: white cell count, ANC: absolute neutrophil count.

Analysis of blood count ratios showed nadir blood count again occurred at week 2–3, a greater relative recovery was seen in the CRT group but was not shown to be significant. Weekly blood count ratios are shown in Fig. 1.

Female gender was not associated with reduced ratios suggesting that although baseline counts are reduced the relative suppression is consistent with that seen in men. T3/4 was associated to reduced nadir WCC and ANC in the IMRT group only: Beta −0.12 and −0.2, SE 0.05 and 0.09, *p* 0.03 and 0.04 for WCC and ANC respectively.

Multiple linear regression analysis did not show dose metrics to be significantly associated with nadir blood count ratios.

Increase in NTCP using parameter values given by Bazan et al. [13] in the IMRT group was statistically significant (*p* < 0.05). Mean NTCP at HT3+ was 0.13 (0.00–0.82) using IMRT relative to 0.07 (0.00–0.93) using CRT. Mean NTCP based on 95% CI parameter estimates were 3–39% in the IMRT group and 4–18% in the CRT group. Comparing against recorded toxicity in this study sample, NTCP modeling well predicted toxicity in the IMRT but predicted toxicity in the CRT group lay marginally outside 95% CI NTCP modeling.

## Discussion

This study looked at PBM doses and blood counts in anal cancer patients receiving concurrent chemoradiation using either IMRT under UK guidelines or CRT per ACT II trial protocol. This study has shown PBM dose to be increased using IMRT. This increase is attributable to the low dose bath associated with IMRT delivery combined with differences in dose regime and PTV delineation. Regression analysis showed the majority of dose metrics to be associated with lower nadir blood counts, with strongest associations seen at 10–15 Gy, and NTCP modeling based on Bazan et al. [13] parameters was predictive of a doubling of risk associated with IMRT. HgB was little suppressed with minimum nadir count 78% of baseline. However, this study does lack data on transfusion; these patients may have received to support HgB. Plats were suppressed but generally speaking not to an extent where HT3+ rates would be a clinical concern. The suppression of WCC and ANC and rates of leukopenia and neutropenia is clinically significant. Weekly treatment response pattern was found to be consistent between groups; in both groups nadir absolute count occurred at week 2–3, this is consistent with responses seen in rectal patients in the US where nadir counts were seen at week 2 [15]. However, neither absolute nor blood count ratios where statistically significant and PBM dose metrics could not be associated to HT3+ rates.

Despite greater mean PBM dose metrics using IMRT and significant association of PBM dose metrics with lower nadir blood counts, actual rates of HT3+ using IMRT were comparable with rates using CRT. This, somewhat paradoxical finding, may be explained by looking at the distribution of PBM dose metrics in the two groups. IMRT PBM doses show a much narrower distribution. A likely hypothesis is that whilst mean PBM dose is higher, doses do not exceed a critical level and therefore IMRT treatment does not significantly impact HT3+ rates under UK practice. This finding is potentially significant given mean PBM dose metrics in the IMRT group were comparable to those reported in the US by Mell et al. [5] (iliac BM V5–20 was 83–56% compared with 85–55%, lumbosacral BM V5–20 was 81–63% compared with 82–66% and lower pelvis BM V5–20 100–94% compared with 99–92%) yet rates were considerably lower at 14% relative to >50%. This is despite 16/21 (76%) of patients exceeding suggested HT3+ dose tolerances for PBM and BM (lumbosacral). One possible explanation is that PBM doses in the Mell study did not share the tight distribution reported in the study, this is supported when looking at the standard deviation of dose metrics in the Mell study which are approximately double those reported here. This, combined with other differences in practice, may explain the much higher rates seen in the US for comparable mean PBM doses.

The distribution of PBM dose metrics may also explain the incorrect prediction by the NTCP model of double the risk on HT3+, but insufficiencies in the NTCP model in this setting maybe a more dominate factor.

On regression analysis, lumbosacral BM doses were either associated with or suggestive of reduced nadir WCC and ANC ratios in both groups. This finding is consistent with findings reported by Mell et al. [5] and Bazan et al. [13] where lumbosacral BM doses were shown to be associated with absolute WCC and ANC count. Strongest associations were seen in iliac BM to reduced nadir WCC, decreasing absolute WCC nadir were reported by Mell et al. but none were found to be statistically significant (*p* > 0.1 in each case). Reducing PBM at the 10–15 Gy level may reduce suppression and subsequent HT3+ rates. Significant sparing of lumbosacral at the 10–15 Gy dose level is challenging due to overlapping structures and the relative position of gross tumour volume. Sparing of iliac BM is more achievable. From univariate regression analysis each percent iliac BM V10–15 can be associated with a relative reduction from baseline of 1% in WCC and 1–2% ANC.

As female gender has been shown to be a significant covariant factor, this study supports any dose tolerances used to guide sparing being gender specific as in Mell et al. [5] n.b. ratio analysis did not show female gender to be predictive of count ratio nadir, as was also found by Yang et al. [15] in rectal patients, showing treatment response is independent of gender.

This retrospective study is limited in its relatively small sample size and the compounding factor of concurrent chemotherapy in determining the precise radiosensitivity of PBM. Subsequently, the results of this work should be interpreted in the context of the concurrent chemotherapy regime and with an awareness of the sample size. Another limitation is that we do not have any information on the function distribution of active bone marrow; FLT-PET has been used to spatially define active morrow [16] and could have indicated the highly proliferative zones of marrow irradiated. However, analysis of both CRT and IMRT patients in this study is a strength in determining the response of PBM to irradiation.

In conclusion, IMRT treatment is associated with a significant increase in dose to PBM relative to CRT treatment. PBM dose metrics are predictive of WCC and ANC suppression in IMRT treatment with each additional percent of iliac/lumbosacral PBM delivered being associated with 1–2% decrease in WCC and ANC. NTCP modeling suggests IMRT results in an approximately doubled risk of HT3+ compared to CRT. Despite this increased PBM dose did not translate into higher rates of HT3+ in this study; the influence of distribution spread of PBM dose metrics on observed HT3+ rates is worthy of further investigation.

## Conflict of interest

None.

## Figures and Tables

**Table 1:** Pelvic bone marrow and sub component dose metrics in conformal and intensity-modulated radiotherapy.

	CRT		IMRT	
	Mean	SD	Mean	SD
*Whole pelvis BM*				
Volume (cm^3^)	1399.6	253.1	1335.6	245.8
V5 (%)	60.4	12.9	89.8	4.3
V10 (%)	57.3	12.5	82.6	4.7
V15 (%)	53.9	12.0	78.7	4.8
V20 (%)	48.7	11.7	73.8	5.2
V25 (%)	46.7	11.7	64.4	7.3

*Iliac BM*				
Volume (cm^3^)	481.7	82.2	453.3	73.5
V5 (%)	40.1	19.5	83.0	7.4
V10 (%)	35.7	17.9	68.7	7.4
V15 (%)	33.2	16.6	61.5	6.8
V20 (%)	30.8	14.7	56.2	5.6
V25 (%)	28.4	14.7	47.5	6.6

*Lumbosacral BM*				
Volume (cm^3^)	317.6	65.1	312.7	62.2
V5 (%)	24.0	24.0	81.1	9.9
V10 (%)	19.8	23.0	71.4	11.5
V15 (%)	17.6	22.6	67.0	11.7
V20 (%)	16.3	22.1	63.1	11.9
V25 (%)	15.1	21.7	59.8	12.1

*Lower pelvis BM*				
Volume (cm^3^)	599.6	119.5	569.6	128.0
V5 (%)	95.9	7.5	100.0	0.0
V10 (%)	94.4	9.1	100.0	0.2
V15 (%)	89.5	9.4	99.0	1.6
V20 (%)	80.0	8.9	93.9	6.6
V25 (%)	77.9	9.0	80.7	11.2

*Abbreviations:* CRT = conformal radiotherapy; IMRT = intensity modulated radiotherapy; SD = standard deviation; BM = bone marrow.

**Table 2:** Blood count baseline and nadir, both absolute and as a ratio of baseline.

		CRT group		IMRT group	
	Baseline bloods	Nadir (absolute)	Nadir (ratio)	Nadir (absolute)	Nadir (ratio)
HgB (g/dl)	12.9 (8.8–16.0)	11.4 (9.2–14.0)	0.89 (0.76–1.00)	10.9 (8.7–14.5)	0.87 (0.63–0.99)
WCC (10^9^/l)	8 (3.4–17.9)	3.1 (1.2–6.6)	0.42 (0.22–0.83)	3.2 (0.8–5.0)	0.38 (0.15–0.62)
ANC (10^9^/l)	5.1 (1.2–14.9)	1.9 (0.4–5.0)	0.42 (0.16–0.97)	2.2 (0.6–3.8)	0.42 (0.15–0.83)
Platelets (10^9^/l)	274.5 (144.0–611.0)	125.4 (44.0–208.0)	0.52 (0.26–0.88)	139.9 (49.0–372.0)	0.46 (0.14–0.77)

*Abbreviations:* CRT = conformal radiotherapy; IMRT = intensity modulated radiotherapy; HgB = haemoglobin; WCC = white cell count; ANC = absolute neutrophil count.

**Table 3:** Univariate linear regression results of pelvis bone marrow dose metrics against absolute nadir blood counts.

		Conformal			IMRT		
Nadir WCC (10^9^/l)			Nadir WCC (10^9^/l)		
		Beta	SE	*p*	Beta	SE	*p*
Iliac BM	V25	−0.044	0.02	0.038	−0.061	0.033	0.086
V20	−0.046	0.02	0.029	−0.047	0.041	0.27
V15	−0.039	0.017	0.036	−0.02	0.035	0.574
V10	−0.035	0.016	0.042	−0.02	0.035	0.574
V5	−0.032	0.015	0.046	−0.036	0.031	0.259

Lower pelvis BM	V25	−0.05	0.033	0.139	0.02	0.021	0.355
V20	−0.054	0.033	0.117	0.03	0.035	0.412
V15	−0.069	0.03	0.03	NA	NA	NA
V10	−0.081	0.03	0.013	NA	NA	NA
V5	−0.089	0.037	0.027	NA	NA	NA
							
		Nadir ANC (10^9^/l)			Nadir ANC (10^9^/l)		
Lower pelvis BM	V15	−0.054	0.024	0.032	0.068	0.121	0.577
V10	−0.064	0.024	0.013	NA	NA	NA
V5	−0.071	0.029	0.024	NA	NA	NA

*Abbreviations:* CRT = conformal radiotherapy; IMRT = Intensity Modulated Radiotherapy; BM = bone marrow.

**Table 4:** Univariate linear regression results of pelvis bone marrow dose metrics against nadir blood counts expressed as a ratio of baseline.

	CRT			IMRT		
Nadir WCC ratio			Nadir WCC ratio		
	Beta	SE	*p*	Beta	SE	*p*
Whole pelvis BM_V25	−0.009	0.004	0.064	−0.003	0.004	0.347
Whole pelvis BM_V20	−0.009	0.004	0.055	−0.008	0.005	0.095
Whole pelvis BM_V15	−0.010	0.005	0.052	−0.014	0.004	0.005
Whole pelvis BM_V10	−0.011	0.005	0.029	−0.015	0.004	0.003
Whole pelvis BM_V5	−0.010	0.004	0.033	−0.015	0.005	0.008

Iliac BM_V25	−0.008	0.003	0.008	−0.005	0.004	0.211
Iliac BM_V20	−0.008	0.003	0.008	−0.012	0.004	0.010
Iliac BM_V15	−0.007	0.003	0.008	−0.011	0.003	0.004
Iliac BM_V10	−0.007	0.002	0.007	−0.010	0.003	0.004
Iliac BM_V5	−0.007	0.002	0.009	−0.010	0.003	0.004

Lumbosacral BM_V25	−0.009	0.005	0.070	−0.006	0.002	0.004
Lumbosacral BM_V20	−0.008	0.004	0.069	−0.006	0.002	0.004
Lumbosacral BM_V15	−0.008	0.004	0.067	−0.006	0.002	0.002
Lumbosacral BM_V10	−0.007	0.004	0.060	−0.006	0.002	0.004
Lumbosacral BM_V5	−0.006	0.003	0.061	−0.006	0.002	0.024

	Nadir ANC ratio			Nadir ANC ratio		
Whole pelvis BM_V25	−0.008	0.005	0.125	−0.004	0.006	0.503
Whole pelvis BM_V20	−0.008	0.005	0.115	−0.011	0.008	0.221
Whole pelvis BM_V15	−0.010	0.005	0.062	−0.020	0.008	0.027
Whole pelvis BM_V10	−0.010	0.005	0.043	−0.021	0.008	0.018
Whole pelvis BM_V5	−0.009	0.005	0.063	−0.020	0.009	0.045

Iliac BM_V25	−0.007	0.003	0.057	−0.003	0.007	0.636
Iliac BM_V20	−0.007	0.003	0.059	−0.013	0.008	0.100
Iliac BM_V15	−0.007	0.003	0.058	−0.013	0.006	0.048
Iliac BM_V10	−0.007	0.003	0.056	−0.012	0.006	0.054
Iliac BM_V5	−0.006	0.003	0.052	−0.011	0.006	0.096

Lumbosacral BM_V25	−0.004	0.004	0.332	−0.009	0.003	0.014
Lumbosacral BM_V20	−0.003	0.003	0.316	−0.009	0.003	0.011
Lumbosacral BM_V15	−0.003	0.003	0.300	−0.009	0.003	0.007
Lumbosacral BM_V10	−0.004	0.003	0.259	−0.009	0.003	0.008
Lumbosacral BM_V5	−0.003	0.003	0.225	−0.009	0.004	0.035

*Abbreviations:* CRT = conformal radiotherapy; IMRT = intensity modulated radiotherapy; WCC = white cell count; ANC = absolute neutrophil count; BM = bone marrow.

## Appendix A Supplementary data

Supplementary Table 1 Patient characteristics and chemotherapy regime.
