# Supplementary material for: Modeling early haematologic adverse events in conformal and intensity-modulated pelvic radiotherapy in anal cancer
Source: Radiother Oncol. 2015 Nov;117(2):246–51. doi: 10.1016/j.radonc.2015.09.009 (PMC4678285; doi:10.1016/j.radonc.2015.09.009)
Supplement: Supplementary Table 1 — Patient characteristics and chemotherapy regime. [file mmc1.docx]

Table One (Supplementary): Patient characteristics and chemotherapy regime.

|  | CRT Group | | IMRT Group | |
| --- | --- | --- | --- | --- |
|  | n | % | n | % |
| Total | 25 | 100% | 21 | 100% |
| Female | 16 | 64% | 14 | 67% |
| Mitomycin + Fluorouracil | 24 | 96% | 15 | 71% |
| Mitomycin + Capecitabine | 0 | 0% | 1 | 5% |
| Mitomycin monotherapy | 0 | 0% | 3 | 14% |
| No Concurrent Chemo | 1 | 4% | 2 | 10% |
| T3-4 | 10 | 40% | 12 | 57% |
| Node + | 6 | 24% | 11 | 52% |
